# Transcriptional Memory in *Taraxacum mongolicum* in Response to Long-Term Different Grazing Intensities

**DOI:** 10.3390/plants11172251

**Published:** 2022-08-30

**Authors:** Yalin Wang, Wenyan Zhu, Fei Ren, Na Zhao, Shixiao Xu, Ping Sun

**Affiliations:** 1 College of Animal Science and Technology, Henan University of Science and Technology, Luoyang 471003, China; 2 Northwest Institute of Plateau Biology, Chinese Academy of Sciences, Xining 810008, China; 3 College of Horticulture and Plant Protection, Henan University of Science and Technology, Luoyang 471003, China; 4 State Key Laboratory of Plateau Ecology and Agriculture, Qinghai University, Xining 810016, China

**Keywords:** transcriptional memory, grazing, *T. mongolicum*

## Abstract

Grazing, as an important land use method in grassland, has a significant impact on the morphological and physiological traits of plants. However, little is known about how the molecular mechanism of plant responds to different grazing intensities. Here, we investigated the response of *Taraxacum mongolicum* to light grazing and heavy grazing intensities in comparison with a non-grazing control. Using de novo transcriptome assembly, *T. mongolicum* leaves were compared for the expression of the different genes under different grazing intensities in natural grassland. In total, 194,253 transcripts were de novo assembled and comprised in nine leaf tissues. Among them, 11,134 and 9058 genes were differentially expressed in light grazing and heavy grazing grassland separately, with 5867 genes that were identified as co-expression genes in two grazing treatments. The Nr, SwissProt, String, GO, KEGG, and COG analyses by BLASTx searches were performed to determine and further understand the biological functions of those differentially expressed genes (DEGs). Analysis of the expression patterns of 10 DEGs by quantitative real-time RT-PCR (qRT-PCR) confirmed the accuracy of the RNA-Seq results. Based on a comparative transcriptome analysis, the most significant transcriptomic changes that were observed under grazing intensity were related to plant hormone and signal transduction pathways, carbohydrate and secondary metabolism, and photosynthesis. In addition, heavy grazing resulted in a stronger transcriptomic response compared with light grazing through increasing the of the secondary metabolism- and photosynthesis-related genes. These changes in key pathways and related genes suggest that they may synergistically respond to grazing to increase the resilience and stress tolerance of *T. mongolicum*. Our findings provide important clues for improving grassland use and protection and understanding the molecular mechanisms of plant response to grazing.

## 1. Introduction

Grassland is a major part of the terrestrial ecosystem, covering 40% of the global land area [1]. It plays an irreplaceable role in human living by regulating the climate, conserving soil and water resources, maintaining biodiversity, and providing biological products [2,3]. The quality of herbage decides the safe production and efficiency of animal husbandry through growth rate, nutritional value, and yield [4,5]. However, herbage has to tolerate several damages in nature, including adverse natural factors, inappropriate use, and human activities, leading to the imbalance of the grassland ecosystem. In particular, herbage needs to tolerate several biotic and abiotic stresses, such as salt [6], drought [7], wounding [8], and foraging [9], because of the geographical distribution and growth characteristics. These factors seriously threaten the survival, growth, production, and value of the grassland vegetation.

In recent years, studies on the response of herbage to external stress (biotic or abiotic stress) and extreme environments have gradually increased, and the point involves many aspects, such as morphological, physiological, and molecular levels [10,11,12]. The research with the emergence of genomic tools and high-throughput phenotyping technologies focuses on investigating the genetic and molecular mechanisms of phenotypic plasticity after different kinds or degrees of stress [13,14]. Phenotype is an important reflection of plant response to plant structure, function, and interactions with the environment, which is influenced by individual traits and the diversity of environmental conditions [15]. Under stress, plants usually exhibit a series of transcriptional responses. For example, a study indicated that cold-stress was related to plant hormone and signal transduction pathways, primary and secondary metabolism, photosynthesis, and members of transcription factors in *Magnolia wufengensis* [16]. Meanwhile, another research identified the metabolic pathways, such as carbohydrate metabolism, photosynthesis, and lipid metabolism, for distinct genotypes in chickpea response to drought and salinity [11]. Plants can acquire the ability to adaptive environmental changes through learning processes, which can be defined as stress memory [17]. Stress memory is influenced by stress times, stress degrees, and different phenological periods of plants [18,19]. A common theme about plant response to a range of biotic and abiotic stresses is the phenomenon of priming whereby previous exposure makes a plant more resistant to future exposure [20]. After priming, plants display a faster or stronger defense response through rapid change in the gene expression if the stress recurs [21]. It also would be reserved for a period of time by the information storage and signal expression in case the harming happens again [22]. The memory span may often be in the range of days to weeks in the acclimated plants, and sometimes may extend to the offspring [21]. The study that concentrated on clonal *Leymus Chinensis* response to long-term overgrazing-induced memory showed that livestock grazing induces a transgenerational effect on growth inhibition through altered gene expression of defense and immune responses, pathogenic resistance, and cell development [23].

Grazing, which is a crucial environmental stress factor, influences plants by repeatedly altering the essentially availability of resources for plant growth [24,25]. The growth conditions, including soil nutrients [2], water [26], and light, are frequently changed by the interaction of foraging, trampling, and fecal accumulation, which further affects herbage growth time [9], productivity [5], palatability [27], and many other aspects. After grazing, leaves were fed to animals with trampling, thus restricting normal growth, which also decreases photosynthesis [28], but increases antioxidant capacity [29] and secondary metabolites synthesis [30]. Long-time grazing plants also perform different resistance strategies to avoid or reduce damage through morphological or biochemical changes, or rapid regrowth/reproduction [31,32]. Meanwhile, herbage usually exhibits dwarfing [23], adjusting the proportion of aboveground and underground [33], and some herbage have low palatability after grazing [27]. The research about transcriptome-wide gene expression plasticity conducted long-term different grazing intensities resulted in gene expression plasticity in the recovery stage of *Stipa grandis*, affecting diverse biological processes and metabolic pathways that were involved in the Calvin–Benson cycle and photorespiration metabolic pathways [13]. Wang et al. researched alfalfa with different grazing tolerance and showed that the DEGs were related to the ribosome and translation-related activities, cell wall processes, and oxygen levels [30]. Although these studies show the transcription process of herbage after grazing, the complex transcription changes that were decided in species were unclear in stress memory during the recovery period of herbage. Therefore, to better understand the transcription changes with different grazing intensities in nature, we focus on the DEGs and metabolism pathways at grazing recovery periods under different grazing conditions.

*T. mongolicum*, one of the main associated species in the alpine meadow, is an edible medicinal herb that is primarily used as an anti-inflammatory, antibacterial, anti-allergy, and antioxidant owing to its bioactive metabolites such as phenolic compounds, polysaccharides, and flavonoids [34]. As a medicinal plant, it has been regarded as a potential substitute for traditional antibiotics in the raising dairy diet in recent years. Li et al. found the supplementation impacts, the kind of ruminal microorganisms, and metabolites that rumen fermentation was enhanced in cows [35]. Therefore, we chose it as the objective to study how transcriptomes respond in natural grazing grassland. We set up three treatments, including light-grazing, heavy-grazing, and non-grazing, and sequenced, assembled, and compared the transcriptomes of *T. mongolicum* to identify gene expression dynamics and DEGs in different grazing intensities. The present study will facilitate further functional genomics studies in *T. mongolicum* and aid in the understanding of the molecular mechanisms that are behind the long-term grazing response in plants and the associated morphological changes.

## 2. Materials and Methods

### 2.1. Plant materials and Treatments

The grazing experiment was performed at the Haibei Alpine Meadow Ecosystem Research Station that is managed by the Northwest Institute of Plateau Biology, Chinese Academy of Sciences. The station is located in the northeastern portion of the Qinghai–Tibet Plateau, China (N37°29′, E 101°12′; 3220 m). This region has a typical continental climate for plateaus, with a short, cool summer and a long, cold winter, and its annual average temperature is −1.7 °C (the maximum and minimum temperatures are 9.9 °C in July and −15.2 °C in January, respectively). The annual mean precipitation is 500 mm, and more than 80% of precipitation occurs during the growing seasons (from May to September). The vegetation in this region mainly consists of *Kobresia humilis Sergiev.*, *Elymus nutans Griseb.*, *Poa pratensis* L., *Carex scabrirostris Kukenth.*, *Gentiana straminea Maxim.*, and *Potentilla nivea* L. The soil has been identified as alpine meadow soil [36].

There were two grazing treatments and fenced treatments that were launched in 2009. The grazing treatments, light-grazing (utilization 30%) and heavy-grazing (utilization 60%), were grazed by sheep for 2 days per month during the vegetation growing season (June-September) every year. The three plots are adjacent to each other, and the terrain and soil conditions are the same, and other influencing factors can be ignored. The vegetation type of the plot is the same as that of natural grassland. In order to identify gene expression responses of *T. mongolicum* in the recovery growth stage after grazing, we chose mid-August for grazing, which was the period of maximum plant biomass in herbage. The new leaves of *T. mongolicum* were randomly collected from three individual plants from 9:00 a.m. to 11:00 a.m. after two weeks of grazing at the beginning of September, and fresh leaves were immediately frozen in liquid nitrogen and then stored at −80 °C. Meanwhile, we collected three biological replicates in the fenced plot as the non-grazing sample.

### 2.2. RNA-Seq Library Preparation and ILLUMNA sequencing

A total of 0.1 g leaves were taken from each plant for total RNA extraction using Trizol reagent (Invitrogen, Carlsbad, CA, USA). The quality and quantity were evaluated using Nanodrop 2000 (NanoDrop, Wilmington, DE, USA), Agilent 2100 Bioanalyzer (Agilent Technologies, Inc, Santa Clara, CA, USA). The RNA samples with a ratio of 260/280 nm greater than 1.8 and RNA integrity number (RIN) > 7 were selected for subsequent processing. The poly(A) mRNA was isolated using Oligo (dT) Beads. The mRNA fragmentation and subsequent RNA-Seq library conversion were carried out using a TruseqTM RNA sample prep Kit (Illumina, San Diego, CA, USA) in accordance with the manufacturer’s instructions. There were nine cDNA libraries that were constructed and each cDNA library was sequenced using the Illumina HiSeq 2000 platform (Shanghai Origingene Bio-pharm Technology Co., Ltd., Shanghai, China).

### 2.3. Quality and De Novo Assembled

The total RNA was extracted from the fresh leaves from 3 treated groups with 3 biological replicates, designated non-grazing (NG1, NG2, NG3), light grazing (LG1, LG2, LG3), and heavy grazing (HG1, HG2 and HG3). Cutadapt (Version 1.16) was used to filter the raw reads, including removing the reads containing adaptor sequences, “N” percentages that were greater than 5%, and low-quality reads (<20% low-quality nucleotides). FastQC (Version 0.11.4) analysis was further performed to estimate the quality of raw reads (www.bioinformatics.babraham.ac.uk/bugzilla/; accessed on 4 December 2019). Then, clean and high-quality reads were do novo assembled into a transcriptome using Trinity software (Version 2.6.6) with three steps (Inchworm, Chrysalis, and Butterfly) [37]. Finally, the results were evaluated by different quality metrics including N50 length of the transcriptome assemblies, the total number of bps in the assembly, and percent of GC.

### 2.4. Functional Annotation and Identification of DEGs

Functional annotation of all the unigenes was conducted by several approaches. First, using the open reading frames (ORFs) process to predict all the assembled transcripts. Then, functional annotation of the transcripts was performed using BLASTX between the unigenes and various protein databases (E-value < 10^−5^) [38], such as the non-redundant protein (NR) database (http://www.ncbi.nlm.nih.gov; accessed on 4 December 2019), the Swiss-Prot protein database (http://www.expasy.ch/sprot; accessed on 4 December 2019), the Kyoto Encyclopedia of Genes and Genomes (KEGG) pathway database (http://www.genome.jp/kegg/; accessed on 4 December 2019), and the Cluster of Orthologous Groups (COG) database (http://www.ncbi.nlm.nih.gov/COG/; accessed on 4 December 2019). The GO distribution for all the unigenes whose expression was significantly altered in the *T. mongolicum* transcriptome were classified using the Blast2GO program [39]. 

The FPKM (fragments per kilo base of exon per million) method was used to determine the expression levels of the unigenes. The DEGs between grazing and non-grazing groups were identified using the edgeR package (FDR < 0.05 and |log_2_FC| ≥ 1) [40]. Correlation analysis was carried out to assess the correlation between the replicates and treatments. For the functional and pathway enrichment analysis, the DEGs were then mapped into GO terms (*p*-value ≤ 0.05) and the KEGG database (*p*-value ≤ 0.05).

### 2.5. RNA-Seq Result Validation by qRT-PCR

A total 10 DEGs were selected to verify our RNA sequencing expression data by qRT-PCR. The primer sequences are listed in Table S1. The Glyceraldehyde 3-phosphate dehydrogenase (GAPDH) gene was used as an internal control to normalize the measured gene expression levels. The total RNA was reverse transcribed into cDNA using the HiScript 1st Strand cDNA Synthesis Kit (Takara, Nanjing, China). Real-time PCR was performed in a real-time PCR platform (CFX96, Bio-Rad, USA) using the AceQ qPCR SYBR Green Master Mix (Takara, Shanghai, China). The cycling conditions were as initial denaturation at 95 °C for 5 min followed by 40 cycles of 95 °C for 10 s and 60 °C for 30 s. For each qPCR analysis, three technical replicates were performed. The 2^−ΔΔCT^ method was used to verify the relative quantity of 10 DEGs in the transcription profile.

### 2.6. Statistical Analysis

The tables in this study were performed using Excel 2020, and the graphs were drawn by GraphPad Prism 8.

## 3. Results

### 3.1. Illumina Sequencing and Reads Assembly

**Figure 1:**
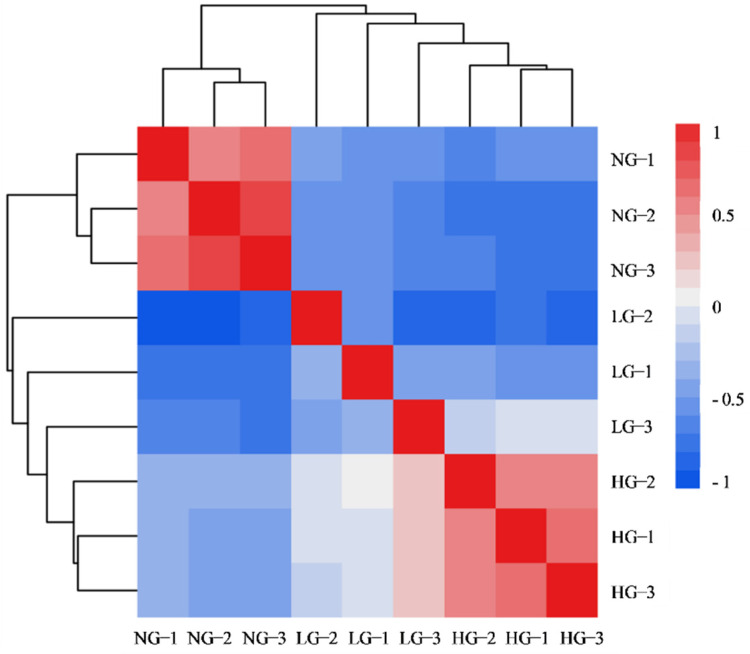
Pearson correlation coefficients of transcript levels in nine samples. NG: non−grazing; LG: light grazing; HG: heavy grazing.

The total raw reads of 377,827,065 from the grazing and non-grazing samples with three biological replicates were obtained by pair-end RNA-Seq sequencing (Table 1). After filtering, 99.8% of the raw reads were retained as clean reads for subsequent transcriptome analysis. All the samples performed high quality results as reflected by all Q20 that were larger than 96.0%. The GC content was approximately 46% in the nine samples. High correlation coefficients were obtained for each treatment, indicating that the data were reliable for further analysis (Figure 1).

**Figure 2:**
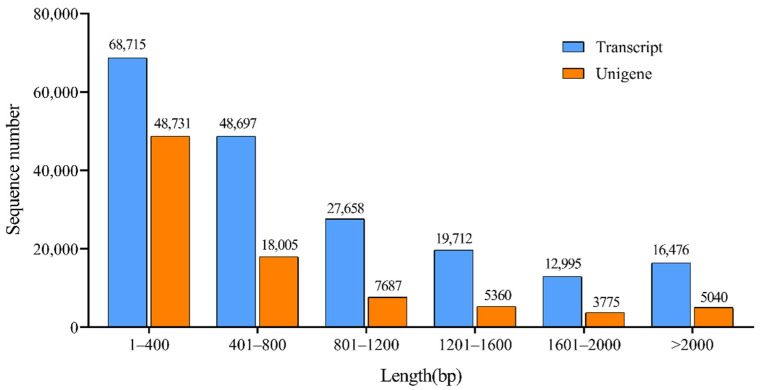
Transcript and unigene length distribution.

A total of 194,253 transcripts were de novo assembled from all paired clean reads in Trinity, with a contig N50 of 1341 bp and average length of 869.48 bp (Table 2). The size distribution of the assembled transcripts showed that 91.52% (177,762) of sequence length ranged from 201 to 2000 bp and the percentage of sequences that were >2000 bp was 8.48% (16,476) (Figure 2). The total number of unigenes of 88,598 was generated. The N50 of the unigenes was 1118 bp, and the average length was 659.36 bp (Table 2). There were 83,558 unigenes with 94.30% in total were <2000 bp in length (Figure 2).

### 3.2. Annotation and Classification of T. mongolicum Unigenes

**Figure 3:**
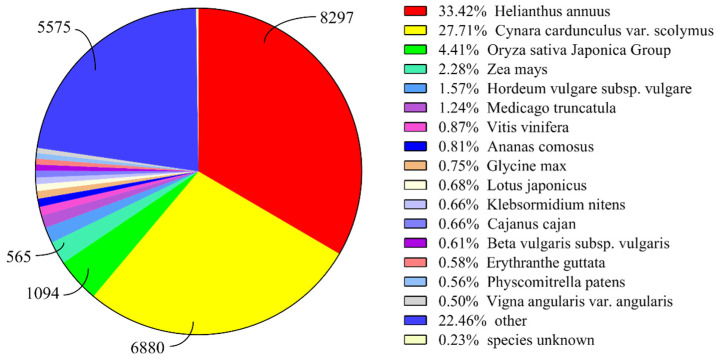
Species distribution of top BLAST hits for each unigene.

To identify the putative function of the *T. mongolicum* unigenes, six complementary methods were utilized. The assembled unigenes were searched against the NCBI non-redundant (Nr), SwissProt, String, GO, KEGG, and Pfam databases using the BLASTX algorithm, with an E-value < 10^−5^. Among these unigenes, 24,825 exhibited significant hits in the Nr database and 16,215 in the SwissProt database. In the other four databases (String GO, KEGG and Pfam), 14,402, 16,039, 10,437, and 10,761 unigenes, respectively, were successfully aligned to known proteins in the nine databases (Table 3). According to the Nr database, the unigene sequences exhibited the most similar BLASTx matches to gene sequences from *Helianthus annuus* (8297), followed by *Cynara cardunculus var. scolymus*(6880) and *Oryza sativa Japonica* Group (1094) (Figure 3).

**Figure 4:**
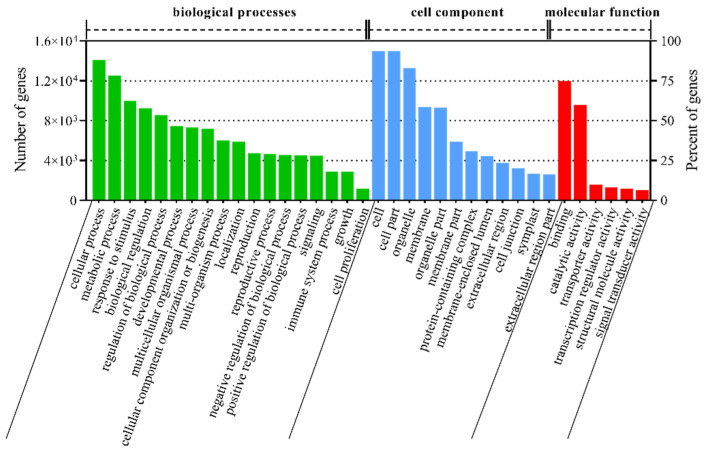
Functional annotation of unigenes based on Gene Ontology (GO) categorization. Green histograms represent “biological process”, blue histograms represent “cell component”, red histograms represent “molecular function”.

The functions of the *T. mongolicum* unigenes were classified via GO analysis. In total, 16,039 unigenes were successfully categorized into 67 functional groups, and these groups were classified into the following three major GO categories using BLAST2GO (Figure 4, only show the unigenes >1000): ‘biological processes’, ‘cell component’, and ‘molecular function’. In the dominant subcategories of the ‘biological processes’ category, ‘cellular process’ (14,083), ‘metabolic process’ (12,536), ‘response to stimulus’ (9991), ‘biological regulation’ (9255), ‘regulation of biological process’ (8546) were the top five groups. ‘Cell’ (14,991), ‘cell part’ (14,984), ‘organelle’ (13,261), ‘membrane’ (9399), ‘organelle part’ (9303) were categorized in the ‘cell component’ category; ‘binding’ (11,972), ‘catalytic activity’ (9553), ‘transporter activity’ (1573), ‘transcription regulator activity’ (1324), and ‘structural molecule activity’ (1170) in the ‘molecular function’ category.

**Figure 5:**
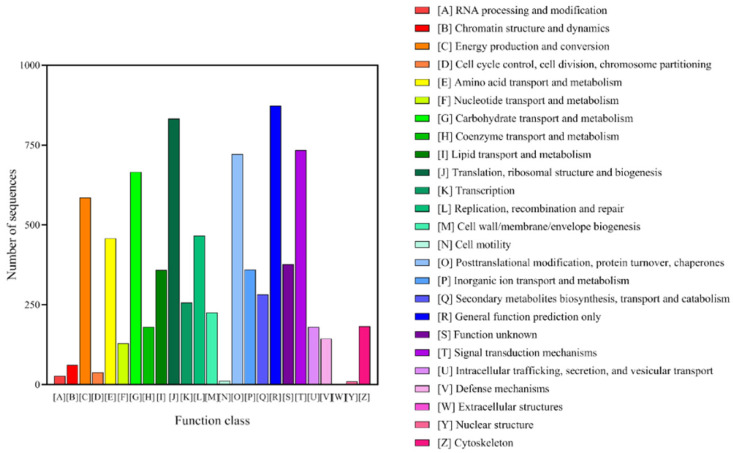
Clusters of Orthologous Group (GOC) classification.

The functions of *T. mongolicum* unigenes were predicted and classified by searching the COG database (Figure 5). Assuming that each protein in the COG database independently evolved from an ancestral protein, we classified the 8168 unigenes based on String annotation into 25 groups of COG classifications. Among these classifications, ‘General function prediction only’ (873, 16.86%) accounted for the largest proportion, followed by ‘Translation, ribosomal structure and biogenesis’ (833, 10.20%), ‘Signal transduction mechanisms’ (734, 8.99%), ‘Posttranslational modification, protein turnover, chaperones’ (721, 8.83%), and ‘Carbohydrate transport and metabolism’ (666, 8.15%). However, few genes were clustered as ‘Nuclear structure’ (10, 0.12%) or ‘Extracellular structures’ (1, 0.01%).

**Figure 6:**
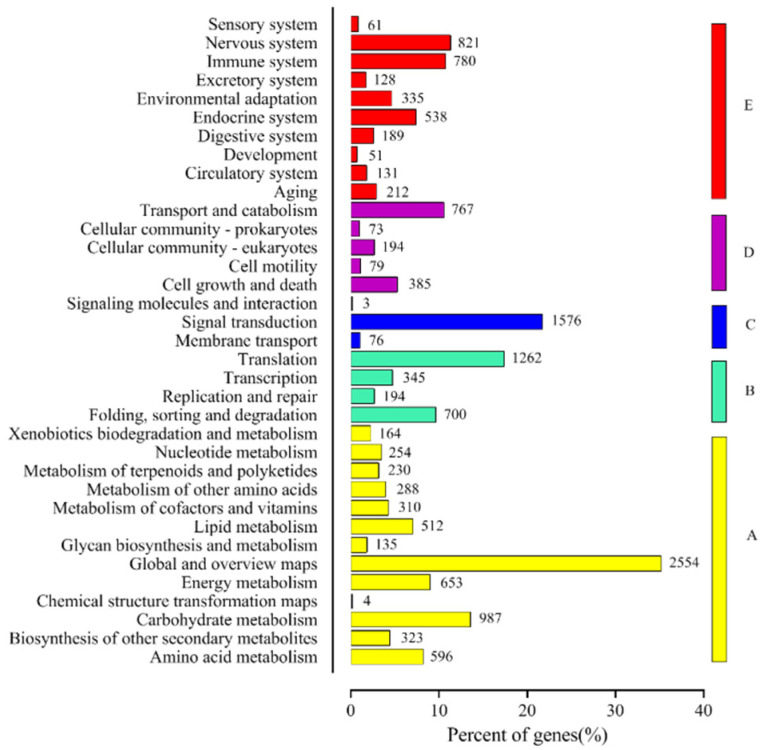
The KEGG metabolic pathway genes that were involved are divided into five branches: A: Cellular Process, B: Environmental Information Processing, C: Genetic Information Processing, D: Metabolism, and E: Organismal Systems.

To identify the active biological pathways in *T. mongolicum*, pathway annotations of the unigenes were performed using the KEGG pathway tool. The KEGG annotated unigenes (12,180) were distributed into five groups with 36 sub-categories (Figure 6). The main groups that were found were ‘Metabolism’ (A, 7010), followed by ‘Genetic Information Processing’ (B, 2501), ‘Environmental Information Processing’ (C, 1655), ‘Cellular Processes’ (D, 1498), and ‘Organismal Systems’ (E, 3246). In these five groups, the most abundant was ‘metabolism’ with 13 sub-groups. ‘Global and overview maps’ (2554) was the highest in these sub-groups, followed by ‘Carbohydrate metabolism’ (987), ‘Energy metabolism’ (653), ‘Amino acid metabolism’ (596), and ‘Lipid metabolism’ (512). ‘Signal transduction’ (1576) and ‘Translation’ (1262) were the largest class in ‘Environmental Information Processing’ and ‘Genetic Information Processing’.

### 3.3. DEGs in Different Grazing Intensity

**Figure 7:**
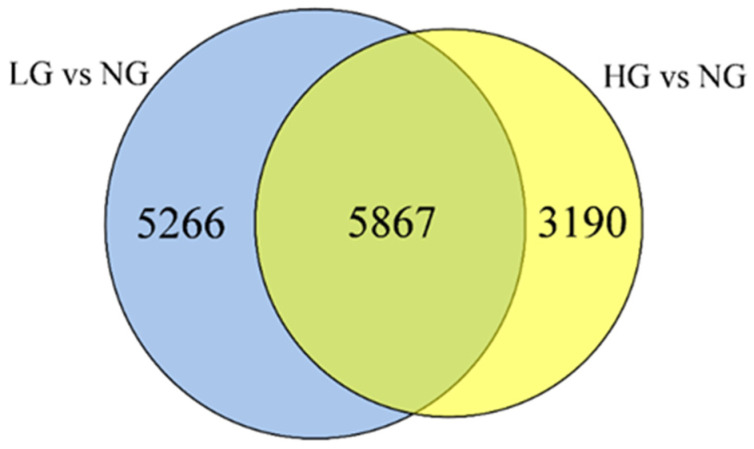
Venn diagram of the functional annotation where each number represents the number of genes in different databases. Blue shows DEGs in light grazing vs non−grazing, light yellow shows DEGs in heavy grazing vs non−grazing, deep yellow indicates co-expression DEGs in two treats.

The DEGs were analyzed relative to non-grazing treatments. A total of 11,134 and 9058 DEGs (*p*-value ≤ 0.05 and |log2 (fold change)| > 1) were identified and analyzed for the LG and HG treatments in our results (Figure 7). In the two treatments, 5867 unigenes were identified that were commonly with induced/repressed, 5266 and 3190 unigenes were induced/repressed exclusively at light grazing intensity and at heavy grazing intensity, respectively.

### 3.4. Pathways Enrichment Analysis of DEGs

Using the KEGG database, pathways displaying significant changes (*p*-value ≤ 0.05) in response to grazing treatment were identified in the two treatments (Table 4). Compared with non-grazing samples, 9 and 15 KEGG pathways were significantly enriched in LG and HG treatments, respectively. The pathways, including ‘Plant–pathogen interaction’, ‘Glycolysis/Gluconeogenesis’, ‘Pentose phosphate pathway’, ‘MAPK signaling pathway-plant’, ‘Starch and sucrose metabolism’, and ‘Circadian rhythm-plant’, were significantly enriched in both grazing treatments, suggesting that plant carbohydrate metabolism and signaling transduction play significant roles in resistance to grazing stress in *T. mongolicum*. The ‘Vasopressin-regulated water reabsorption’ was only enriched in LG treatment; ‘Photosynthesis-antenna proteins’, ‘Calcium signaling pathway’, ‘Biosynthesis of secondary metabolites’, ‘Carbon fixation in photosynthetic organisms’, and ‘Plant hormone signal transduction’ were only significantly enriched in the HG treatment, meaning that the process of resistance to grazing in *T. mongolicum* was complex. The pathway of ‘Biosynthesis of secondary metabolites’ exhibited the most DEGs, suggesting that the HG treatment resulted in more secondary metabolites compared with LG treatment in *T. mongolicum*.

### 3.5. Validation of Gene Expression Profiles by qRT-PCR

**Figure 8:**
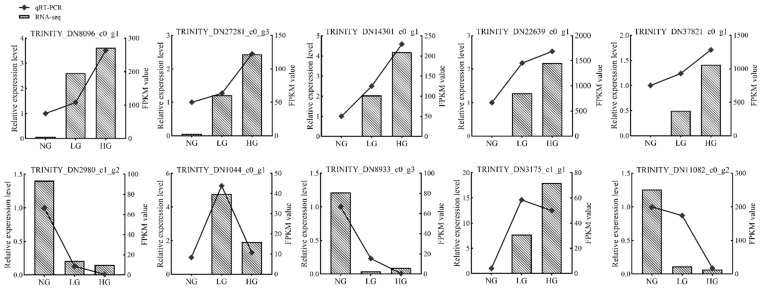
qRT-PCR validation of the RNA-Seq results of *T. mongolicum* in response to grazing.

To confirm the accuracy and reproducibility of this RNA-Seq data, 10 DEGs were chosen randomly for qRT-PCR with two grazing treatments, including seven up-regulated genes and three down-regulated genes in the unigene dataset (Figure 8). All but one of the 10 genes in the two treatments had similar trends in expression patterns in the qRT-PCR assays as in the RNA-Seq data, confirmed the reliability of our RNA-Seq data.

### 3.6. Identification of Signal Transduction-Related Unigenes

According to KEGG enrichment analyses of the DEGs, signal transduction in plants was evidently influenced by grazing stress. In the pathway of ‘MAPK signaling pathway-plant’, we found four mitogen-activated protein kinase (MAPKs) genes were up-regulated in two treatments, which play important roles in signal transduction that is induced by abiotic stress. Meanwhile, we found more than 240 genes were predicted to encode protein kinases with varying expression levels in plants under grazing-treatment conditions (Table S1). There are 110 genes that were common in the two treatments compared with NG. A total of 32 genes and 28 genes were concluded in the group of serine/threonine-protein kinases and receptor-like protein kinase (RLKs) with most being up-regulated, and 11 genes belonging to RLKs in the group of serine/threonine-protein kinases. We also found two wall-associated receptor kinases and two cysteine-rich receptor-like protein kinases in the RLKs group. There are further three Ca^2+^-related protein kinases, seven ATP binding-related kinases, and three pyruvate kinases in all the protein kinases.

Calcium, a metal ion, is a secondary messenger in plant cells and plays essential roles in many signaling pathways. The calcium binding proteins of the EF-hand super-family are associated with the regulation of all aspects of cell function. In our data, in total 42 genes were predicted to encode Ca^2+^-related proteins with most of the genes being up-regulated compared with NG in different treatments (Table S1). We identified five calcium-binding EF-hand proteins (CBL), five calcium-dependent protein kinases (CDPKs) and their related proteins, and two sodium/calcium exchanger-related proteins in two treatments. Meanwhile, we found three CBL-interacting protein kinases (CIPKs) were unique in the LG treatments.

The DEGs that were related to reactive oxygen species (ROS) metabolism were found in 39 genes in two treatments. It included peroxidase (POD), catalase (CAT), glutathione S-transferase (GST), glutaredoxin, and thioredoxin, which play important roles in ROS scavenging. A total of 16 co-expressing DEGs were found in two treatments with 13 up-regulated, while 16 DEGs were only in the LG treatment with 12 up-regulated and 7 DEGs were only in the HG treatment with 4 down-regulated, which means that the LG treatment had more activated scavenging (Table S1).

### 3.7. Identification of Plant Hormone-Related Unigene

Plant hormones play a major role in plant growth and development and regulate various metabolic processes. EREBP, the most downstream element in the ethylene signal transduction, was from AP2/ERF transcription factor superfamily. We found 14 genes about EREBP in two treatments with five genes down-regulated and nine genes that were up-regulated, while interestingly four genes only in LG were all up-regulated and five genes were down-regulated with five out of six in HG, exhibiting ethylene may express in different ways in the two treatments (Table S2). Auxin closely interacts with ethylene, thus jointly regulating many biological processes in plants. We found 30 genes in two treatments. There were 13 genes that were discovered in two treatments with four genes that were down-regulated and nine genes that were up-regulated. There were five SAUR and one IAA and one auxin response factor (ARF) that were included in the 13 genes, which were important proteins in the regulatory process of auxin. In addition, LG and HG treatments also included several genes that were involved in auxin-related genes, and most of the genes were up-regulated, meaning that the expression of auxin had increasing trends after grazing.

The genes, which are associated with the biosynthesis of the defense-type hormone jasmonic acid (JA), were found in two treatments with 13 genes, including one lipoxygenase (LOX), one 12-oxophytodienoate reductase, two allene oxide cyclase (AOC), eight Acyl-CoA-related proteins, and one fatty acid desaturase (FAD), and generally interacted in JA biosynthesis (Table S2). In these genes, the majority of these genes were up-regulated, except the 12-oxophytodienoate reductase and three Acyl-CoA-related genes. We also found three LOX and one allene oxide synthase (AOS) with high expression exclusively in HG, which may mean that high intensity grazing had a significant effect on plant growth and defense.

### 3.8. DEGs Involved in Metabolism and Biosynthesis

Based on the KEGG pathway enrichment analyses, many DEGs are associated with metabolism and biosynthesis. We found that most of the enriched pathways were included in carbohydrate metabolism. Compared with NG treatments, the crucial enriched pathways in LG and HG were “Starch and sucrose metabolism”, “Pentose phosphate pathway”, and “Glycolysis/Gluconeogenesis” (Table S3). In the “Starch and sucrose metabolism” pathway, we found a total of 16 co-expressing DEGs in LG and HG treatments with 13 up-regulated genes, exhibiting a trend that converting to sucrose and other forms of sugar. We found a starch synthase gene (TRINITY_DN17902_c0_g1) was decreased by 6.9-fold, while an alpha-amylase gene (TRINITY_DN4625_c0_g1) was up-regulated compared with NG by 6.6-fold. Meanwhile, genes that were involved in the monosaccharide conversion and hydrolysis process were obviously up-regulated, meaning that glucose metabolism was activated after two weeks of grazing. In the “Glycolysis/Gluconeogenesis” pathway, we found a total of 20 co-expressing DEGs with 18 up-regulated genes. One pyruvate dehydrogenase, 3 pyruvate kinase, and 4 alcohol dehydrogenase had higher expression compared with NG, which means the “Glycolysis/Gluconeogenesis” process in our results mainly carried out the glycolysis process after grazing. Meanwhile, three phosphoglycerate-related proteins were found, which also confirmed the occurrence of glycolysis in *T. mongolicum*. “Pentose phosphate pathway” was found in 11 co-expressing genes that were up-regulated. One transketolase gene, which plays an important catalytic role in pentose biosynthesis, had a high expression compared with NG by 10.1-fold. One carbohydrate kinase and one glucose-6-phosphate 1-dehydrogenase were also noted in this pathway which was up-regulated by 7.4-fold and 6.1-fold, respectively. There were four fructose phosphate-related proteins with up-regulated expression that were common in the “Pentose phosphate pathway” and “Glycolysis/Gluconeogenesis” pathway. One phosphoglucomutase and one glucose-6-phosphate isomerase, the principal enzymes of glucose metabolism were present in all three pathways and were up-regulated 9.9-fold and 4.4-fold, respectively.

The “Biosynthesis of secondary metabolites” pathways were significantly enriched in the HG treatment without LG treatment. High intensity grazing resulted in secondary-related genes rapidly rising and total 188 genes that were found to be involved in a number of pathways, which means that high intensity grazing may have an important effect on plant resistance against outside stress (Table S3). The main concerned pathways of secondary metabolism were phenylpropanoid biosynthesis. The phenylpropanoid-related protein, including three phenylalanine ammonia-lyase, one 4-coumarate—CoA ligase, and three polyphenol oxidase, which are key enzymes of phenylpropanoid pathways, had high expression compared with NG, were down-regulated. We also found several genes that were involved in shikimate pathways, which are the precursor to synthesize phenylalanine. The numbers of secondary metabolism-related proteins, including peroxidase, pyruvate kinase, alcohol dehydrogenase, isopentenyl diphosphate isomerase, glyoxylate/hydroxypyruvate reductase, and linoleate 13S-lipoxygenase, were also up-regulated with high expression. The protein family of cytochrome P450 was found in a total of 42 genes with the most of up-regulation, which not only acted on secondary metabolism but also played an important role in multiple metabolic processes of the plant.

### 3.9. Identification of Photosynthesis Involved in the Response to Grazing Stress

**Figure 9:**
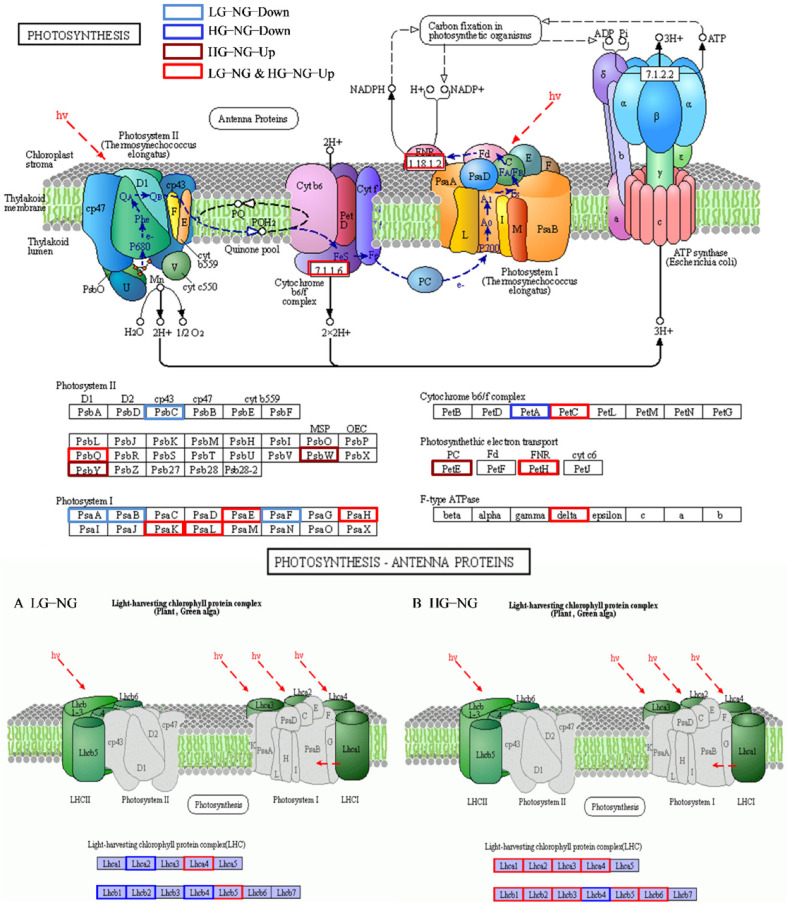
The DEGs about photosynthesis pathway and photosynthesis-antenna proteins in two grazing treatments. Blue blocks mean down-regulated genes in light grazing treatment vs non-grazing treatment, dark blue blocks represent down-regulated genes in heavy grazing treatment vs non-grazing treatment, dark red blocks indicate up-regulated genes in heavy grazing treatment vs non-grazing treatment, red blocks mean up-regulated co-expression genes in light grazing treatment vs non-grazing treatment and heavy grazing treatment vs non-grazing treatment.

Grazing caused a serious loss of plant leaves, which was bound to affect photosynthesis. Depending on our results, we identified many DEGs that were related to photosynthesis and chloroplast. KEGG analysis also showed the pathways of photosynthesis were enriched in the HG treatments, including “Photosynthesis-antenna proteins” and “Carbon fixation in photosynthetic organisms”. The “Photosynthesis-antenna proteins” pathway was the top enriched pathway in HG, which means the photosynthesis-related process had an intense change in the HG treatments of *T. mongolicum*. We found a total of 24 DEGs that were related to the photosynthesis pathway, including photosystem I (PSI), photosystem II (PSII), ferredoxin, oxygen-evolving enhancer protein, and cytochrome b6-f protein. There were also 10 genes jointly in LG and HG that were all up-regulated with high expression (Table S4). Interestingly, five out of six genes that were related to PSI and PSII were down-regulated in the LG treatment, while the related genes in HG treatment had an opposite trend and were up-regulated. A total of 22 genes that were involved in the “Photosynthesis-antenna proteins” pathway were found in our result with 4 and 12 genes that were only classified in LG and HG, respectively (Figure 9). The main protein of this pathway, chlorophyll A-B binding protein, had a similar tendency to be down-regulated in LG compared with NG, while nearly all the proteins were up-regulated with high expression in the HG treatment, which may mean the different responses of plant resistance to different grazing intensities. Most photosynthesis-related proteins and photosynthetic pigment genes were up-regulated compared with NG, which may mean that *T. mongolicum* significantly increased the capacity of photosynthesis to balance plant growth after two weeks of grazing.

## 4. Discussion

The Qinghai-Tibet Plateau was famous for its special geographical location and highly vulnerable climate. Over the last decades, most of the areas have been grazed by Tibetan sheep and yaks at different intensities. Grazing is the major factors affecting the ecosystem through long-term changes including vegetation loss. Many studies were focused on the influence of vegetation through different grazing intensities [41,42], but, as the transcriptome RNA-Seq analysis developed, some had concern about the RNA-level effects on vegetation by grazing [23,43]. While, prior to this study, grazing was a complex stress for plants with biotic and abiotic effects resulting in the mechanisms of grazing that are not clear yet, most scholars have paid attention to the effect of stress with design and treatment in the lab but not in herbage/plants in natural habitat. The research on wild conditions about how grazing influences plant transcriptomes was lacking, so we chose *T. mongolicum*, a typical species that is found in the grassland of the Tibet Plateau, as our research object, and using transcriptomic RNA-Seq analysis, to probe the molecular mechanisms that were influenced by different grazing intensity. This report obtained new insights into the molecular mechanisms of the grazing stress response in *T. mongolicum*.

For all the samples from the treatment plants, more than 37 million high-quality reads were obtained, which were de novo assembled into 88,598 unigenes with an N50 of 1118 bp and the average length of the assembled unigenes was 659 bp, indicating that the assembly was of high quality. Among the functional annotations, 24,825, 16,039, and 10,437 genes were annotated with the NR GO, and KEGG databases, respectively. We found a total of more than 14 thousand DEGs in LG and HG traits, but only 5867 genes existed in the two treatments. Most of the genes were especially distributed in LG or HG traits, which indicated the molecular mechanisms of *T. mongolicum* to respond to stress by grazing were complex and discrepant.

### 4.1. Signal Mediate Responses under Different Grazing Intensities

Under stress, plants can trigger the expression of genes that are involved in multiple signal transduction pathways and further activate downstream regulatory pathways that are associated with physiological adaptation. Ca^2+^, as an important second messenger in plants, which is changed by nearly all signals about developmental, hormonal, and stresses [44,45], was activated in *T. mongolicum* in response to the leaf loss due to grazing. In our results, we found several genes relating to Ca^2+^-related proteins. The expression of the main proteins, such as calcium exchanger-related protein, calcium-binding EF-hand proteins (CBLs), calcium-dependent protein kinases (CDPKs), and CBL-interacting protein kinases (CIPKs), were all up-regulated after grazing. Both CBLs and CIPKs, as the key step of Ca^2+^ signal transduction, belonged to the group of serine/threonine protein kinases, that interact and regulate plant responses to various environmental stress [46,47]. After being stimulated by biotic and abiotic stress, plants form specific Ca^2+^ signals in the cells that directly binds to the EF-hand domain to change the conformation of CDPKs with kinase active sites that are exposed and kinase activity is activated, which play a key role in decoding Ca^2+^ signatures and transducing signals [44]. CDPKs also can interact with different kinds of pathways to control plant growth and development, hormone signal transduction, and adaptation to stress [45]. A previous study also found that CDPKs and MARKs could interact to mediate signal transduction in wound-induced ethylene production [48]. In our results, we found four MAPKs in two treatments that were enriched to the “MAPK signaling pathway”. The MAPK signaling pathway is considered to act on a variety of biological processes in plants, including the transcriptional activation of defense genes, the synthesis of plant resistance-related hormones, and the outbreak of ROS that is induced by pathogens and the thickening of cell walls [49,50].

Besides, we also found other kinds of protein kinases, more than 250, which may play an important role in the perception of grazing in *T. mongolicum*. RLKs, one kind of important plant protein kinase that is located in the cell membrane, could receive stimulus signal and participated in intracellular signal transduction processes as the receptor of signaling molecules [51]. In our result, we mainly found LRR-RLKs, wall-associated receptor kinase, and cysteine-rich receptor-like protein kinase to participate in the signal transduction to defend the effect of grazing. We also found several ATP binding-related kinases, carbohydrate kinases, and pyruvate kinases, which may act on the energy transfer and secondary metabolism process of *T. mongolicum* during adaption of grazing.

ROS are not only normal byproducts of plant cellular oxidative metabolism, but also well-known secondary messengers in various cellular processes in plants. Studies have found that ROS had a dual function in plants, and the transformation depends on its concentration: it could participate in the regulation of plant growth and development and response to adversity stress, as the important signal molecule, in low concentrations; it could become a cell killer when the concentration exceeds the limit that the cell can withstand [52]. Genes encoding POD, GST, GPX, and PPO were identified with up-regulated expression in our results, which means *T. mongolicum* activated the ROS system to defend against damage after grazing.

### 4.2. Phytohormone Signals under Different Grazing Intensities

Plant hormones play key roles during stress through the interaction of multiple hormones and coordination with various signal transduction pathways [53]. We found that grazing mainly regulated three hormones, auxin, JAs, and ethylene, to adapt to the stress and sustain growth in *T. mongolicum*. Ethylene plays a major role in plant growth and development and response to biotic or abiotic stresses [54]. In our results, we found several genes relating to EREBP and ERF. As one of the plant-specific transcription factors, the ERF subfamily transcription factors are key regulators downstream of the ethylene-mediated stress response signal pathway [8]. But interestingly, most of the genes that were only involved ERF in HG were up-regulated, while in LG they were down-regulated, which may mean different grazing intensities changed the ethylene expression level to adapt to the environment. Ethylene could also interact with auxin to regulate plant growth and development. Auxin affects plant development, coordinates leaf senescence, and participates in multiple signaling pathways with other hormones [55]. The ARF is considered to be a key protein that directly affects auxin downstream response genes. Studies have shown that ARF19 is a positive auxin signal regulating factor, and its expression increases with leaf senescence [56]. We found one ARF19 gene (TRINITY_DN12575_c0_g1) in two treatments had up-regulated expression by 4.86-fold, which may mean that after grazing *T. mongolicum* decreased senescence and increased leaf growth. We also found two IAA proteins and five SAUR proteins with up-regulated transcripts, which jointly participated in the regulation of synthesis and transport to affect the plant growth and signal transduction.

JA signal pathways were the other essential pathways in our results to defend against stress. The response of jasmonic acid to plant defense is very rapid after being injured or eaten by herbivores. It immediately activates the biosynthesis of jasmonic acid and initiates downstream genes through signal transduction, such as inhibiting the digestion process of the eaten leaves by producing defensive molecules [57,58]. We found several genes in Acyl-CoA protein, FAD, AOC, and LOX, which were key enzymes in JA signal pathways. Meanwhile, JAs also could regulate the secondary metabolic process and synthesize the secondary metabolites to resist the harm that is caused by external pressure [59]. However, more and more studies have shown that activating the JA pathway in plants can reduce the activity of plant cell cycle proteins and block the cell cycle, thereby weakening the cell division and elongation of plants, and slowing plant vegetative growth [60]. Some studies have also shown that the resources that are available to the plant and its ecological environment are limited, and growth and defense will consume these limited resources by competing and promoting each other [61]. While the resources that are accumulated by plant growth can be used for defense, and the resources that are recovered by the defense can be used for plant growth [61]. Therefore, under natural conditions, in order to better adjust to the external environment, plants must “balance” between growth and defense to keep the two in a balanced state [28]. As the most typical hormone of growth and defense, ethylene, auxin, and JAs showed different expression levels in our results after different grazing treatments, which adjusted the *T. mongolicum* growth and defense to adapt to external stress.

### 4.3. Metabolism and Biosynthesis under Different Grazing Intensities

In the process of plant growth and metabolism, carbohydrates are not simply a product of plant photosynthesis, but also a substrate for respiration, providing carbon framework and energy for plant growth and development, and enhancing plant resistance to stress [62]. Carbohydrate metabolism is the center of the entire biological metabolism, connecting the metabolism of proteins, lipids, nucleic acids, and secondary substances [63]. Especially, when plants lose lots of leaves after grazing, which both source and sink of carbohydrates, carbohydrate metabolism became a crucial process to maintain biological functions and adapt to environmental stress. In our result, we found several pathways of carbohydrates metabolism, including “Starch and sucrose metabolism” “Pentose phosphate pathway”, and “Glycolysis/Gluconeogenesis”, were all enriched in LG and HG based on KEGG analysis. In the “Starch and sucrose metabolism” process, we found carbohydrates tended to convert to sucrose and other monosaccharides, increasing the content of soluble sugars in *T. mongolicum*. Studies have shown that soluble sugars could increase plant stress resistance and regeneration rate to regulate growth [64]. In the “Glycolysis/Gluconeogenesis” pathways, we found several genes that were involved in glycolysis with up-regulated expression, such as glyceraldehyde-3-phosphate dehydrogenase, pyruvate kinase, phosphoglycerate kinase, enolase, aldolase, and hexokinase, participating in the important process of glycolysis. The pyruvate kinase and hexokinase were two of the three irreversible enzymes in the glycolysis process that significantly affect the direction of sugar metabolism [65]. We also found several alcohol dehydrogenases were enriched in this pathway which were up-regulated, which was the key enzymes of anaerobic respiration [66]. This means that *T. mongolicum* had an anaerobic process in sugar metabolism. Meanwhile, alcohol dehydrogenase is also regulated by mechanical injury and disease resistance, and it has an obvious connection with secondary metabolism in plant resistance [67,68]. In “Pentose phosphate pathway”, all genes that are enriched in these pathways were up-regulated, and few of genes were the same as “Glycolysis/Gluconeogenesis” and “Starch and sucrose metabolism”. There were three pathways that interacted and regulated the carbohydrate metabolism of *T. mongolicum* to adapt to external environment change.

The secondary metabolism plays a significant role in defense against external adversity and increases defense ability after plant injury [69]. Facing feeding or injury of animals and humans, plants increase the secondary metabolites by changing the physiological and biochemical metabolic pathways, forming a large number of compounds such as terpenes, phenols, and alkaloids, increasing their own chemical defense capabilities [70]. Meanwhile, damage is also caused phenylalanine ammonia lyase (PAL), peroxidase, glucanase, ubiquitin protease system, and transcription factor response [59]. In our results, we found several genes in the phenylalanine metabolic pathway with significantly up-regulated expression. The increased activity of PAL is conducive to the synthesis of lignin, flavonoids, and other secondary metabolites, and it improves plant resistance in terms of disease resistance and stress resistance [71]. Glucosinolates that were produced in the metabolic pathway of phenylalanine are also markedly induced in mechanical damage and can resist the destruction of herbivores [72]. Early studies also have found that the total phenol content in the leaves of damaged plants has increased dramatically [71]. In our results, we found a number of the genes of polyphenol oxidase in HG treatment, which may mean that heavy grazing will result in high defense ability. Previous research has suggested that after the wound-induced damage, plants could form a sterile wound skin to protect the underlying tissue from drying out and resist the invasion of pathogens, and the cell wall matrix at the wound site needs polyphenols and fatty compounds for nitrosation to make the wound heal [73]. In total, *T. mongolicum* was increasing defense ability through phenylalanine metabolism and increasing healing speed through polyphenols synthesis.

### 4.4. Photosynthesis under Different Grazing Intensities

Photosynthesis is the basic physiological process underlying plant growth and development, but grazing can significantly affect this process. Previous research showed that after grazing, photosynthesis was inhibited for a few days, subsequently, it would increase by compensatory photosynthesis [74]. Genes of porphyrin and chlorophyll metabolism, participating in chlorophyll synthesis, were up-regulated in two treatments, implying that the photosynthesis increased after two weeks of grazing. In our research, however, we found the gene expression level that was related to photosynthesis was different. For instance, the HG treatment had more significantly increased expression than the LG treatment with almost all single genes being up-regulated in HG while down-regulated in LG. This mainly includes the expression of genes that are involved in PSI, PSII, and light-harvesting chlorophyll proteins. The efficiency of photosynthesis is closely connected with the activities of PSI and PSII, converting light energy to chemical energy via electron transport [75]. Light-harvesting chlorophyll protein is the main photoreceptor in the plant photosynthesis process [76]. A total of 10 co-expressing genes in two treatments were up-regulated, while eight genes in HG were up-regulated and six genes in LG were down-regulated, which means that the photosystem functions were increased after two weeks of grazing and the compensatory photosynthesis process was more activated in the HG treatment. Furthermore, the photosynthesis process could adjust the generation of ROS, and thus affect the efficiency of photosynthesis [77]. In our research, the scavenging oxidase was up-regulated, which means that after two weeks of grazing, the plant photosynthesis system recovered gradually.

## 5. Conclusions

After grazing, various metabolic and regulatory processes are altered to adapt to grazing injuries, and these processes often do not disappear immediately but are accompanied by a long stage of plant growth. In this study, we provide a comprehensive description of the transcriptomic responses of plants under natural grazing conditions, and found the genes that were related to the changes in signal and hormone transduction mechanism, carbohydrate metabolism, and photosynthesis in plants after grazing for 2 weeks at maximum biomass. Based on the transcriptomic data, grazing may first stimulate induced signal transduction, including Ca^2+^, phytohormone, and ROS signaling. All signaling pathways act on protein kinases that can switch on numerous proteins. Then, several metabolism pathways were activated to reduce the damage from leaf loss after grazing through accelerating carbohydrate metabolism and increasing the synthesis of secondary metabolites. The photosynthesis process was also significantly improved to accumulate photosynthetic products and scavenge excess ROS. Meanwhile, the *T. mongolicum* had a stronger response in the photosynthesis process and secondary metabolism, which may mean a compensatory growth in high intensity grazing than light intensity grazing. The proposed model may facilitate future studies on the molecular mechanisms underlying grazing stress responses in plants.

## Figures and Tables

**Table 1:** Sequencing the *T. mongolicum* transcriptome in nine samples from plants that were light grazing treated (LG−1, LG−2, LG−3), heavy grazing treated (HG−1, HG−2, HG−3), and non−grazing−treated (NG−1, NG−2, NG−3).

Sample	Raw Reads	Clean Reads	Clean Assembled Bases	Q20 (%)	GC (%)
LG−1	34,877,558	34,826,890	5,179,996,953	96.94	46.51
LG−2	37,462,782	37,404,946	5,562,407,266	96.81	46.48
LG−3	42,165,774	42,080,700	6,247,968,369	96.94	46.02
HG−1	50,461,110	50,385,728	7,483,079,145	97.13	48.55
HG−2	41,660,330	41,560,876	6,177,856,152	96.56	47.49
HG−3	44,534,628	44,395,988	6,593,904,050	96.9	48.35
NG−1	38,969,368	38,908,870	5,795,654,929	97.06	48.16
NG−2	39,309,852	39,241,864	5,835,824,125	96.91	46.58
NG−3	48,385,668	48,308,606	7,164,002,702	97.24	46.76
All−unigene	377,827,065	377,114,472	56,040,693,687	96.94	47.21

**Table 2:** Statistics of sequencing and assembly results.

Type	Unigene	Transcript
Total sequence number	88,598	194,253
Total sequence base	58,417,702	168,900,058
Largest length (bp)	10,562	10,562
Smallest length (bp)	201	186
Average length (bp)	659.36	869.48
N50 length (bp)	1118	1341
N90 length (bp)	259	371

**Table 3:** Annotation statistics of *T. mongolicum* unigenes.

Type	Unigene	Transcript
Nr	24,825	69,093
SwissProt	16,215	43,153
String	14,402	35,730
GO	16,039	42,629
KEGG	10,437	28,358
Pfam	10,761	33,095

**Table 4:** Significantly enriched gene pathways involving differentially expressed genes (DEGs) following the grazing treatment.

ID	Pathways	Q-Value	No. of Genes
LG vs. NG			
ko04626	Plant–pathogen interaction	0.001	20
ko04962	Vasopressin-regulated water reabsorption	0.003	6
ko00010	Glycolysis/Gluconeogenesis	0.008	19
ko00030	Pentose phosphate pathway	0.010	11
ko04016	MAPK signaling pathway-plant	0.019	23
ko03018	RNA degradation	0.022	21
ko00511	Other glycan degradation	0.033	6
ko00500	Starch and sucrose metabolism	0.034	16
ko04712	Circadian rhythm-plant	0.050	11
HG vs. NG			
ko00196	Photosynthesis-antenna proteins	0.000	10
ko00500	Starch and sucrose metabolism	0.003	17
ko00010	Glycolysis/Gluconeogenesis	0.004	18
ko04016	MAPK signaling pathway-plant	0.008	22
ko04020	Calcium signaling pathway	0.017	7
ko04712	Circadian rhythm-plant	0.019	11
ko04626	Plant–pathogen interaction	0.021	16
ko00680	Methane metabolism	0.022	10
ko04922	Glucagon signaling pathway	0.022	10
ko01110	Biosynthesis of secondary metabolites	0.030	128
ko04391	Hippo signaling pathway-fly	0.041	5
ko04915	Estrogen signaling pathway	0.041	5
ko00030	Pentose phosphate pathway	0.042	9
ko00710	Carbon fixation in photosynthetic organisms	0.047	13
ko04075	Plant hormone signal transduction	0.051	18

## Data Availability

Not applicable.

## Supplementary Materials

The following supporting information can be downloaded at: https://www.mdpi.com/article/10.3390/plants11172251/s1, Table S1: The primer sequences in qRT-PCR; Table S2: Ca^2+^- and ROS-related DEGs and protein kinases under different grazing intensities; Table S3: DEGs that were associated with plant hormone under different grazing intensities. Table S4: DEGs that were related to metabolism and biosynthesis under different grazing intensities. Table S5: DEGs that were associated with photosynthesis under different grazing intensities.
